# BIOF–HILO Assay: A New MALDI–TOF Mass Spectrometry Based Method for Discriminating Between High- and Low-Biofilm-Producing *Candida parapsilosis* Isolates

**DOI:** 10.3389/fmicb.2019.02046

**Published:** 2019-08-30

**Authors:** Elena De Carolis, Silvia Soldini, Marilisa La Rosa, Fabio Nucci, Brunella Posteraro, Maurizio Sanguinetti

**Affiliations:** ^1^Dipartimento di Scienze di Laboratorio e Infettivologiche, Fondazione Policlinico Universitario Agostino Gemelli IRCCS, Rome, Italy; ^2^Dipartimento di Scienze Gastroenterologiche, Endocrino-Metaboliche e Nefro-Urologiche, Fondazione Policlinico Universitario Agostino Gemelli IRCCS, Rome, Italy; ^3^Istituto di Patologia Medica e Semeiotica Medica, Università Cattolica del Sacro Cuore, Rome, Italy; ^4^Istituto di Microbiologia, Università Cattolica del Sacro Cuore, Rome, Italy

**Keywords:** MALDI-based assay, *Candida parapsilosis*, biofilm formation, candidemia, composite correlation index

## Abstract

*Candida parapsilosis* is the most frequent cause of catheter-related candidemia among non-*Candida albicans* species. This may be related to intrinsic capabilities as adhering and forming a biofilm on abiotic surfaces such as on medical devices. As previously demonstrated, patients infected with high biofilm-producing *C. parapsilosis* isolates had a greater mortality risk compared to patients infected with low biofilm-producing *C. parapsilosis* isolates. We developed the BIOF–HILO assay, a MALDI–TOF mass spectrometry (MS)-based assay, which compares mass spectra obtained from attached and suspended isolate cells during the early (i.e., 3-h) adhesion phase of *in vitro* biofilm formation. The composite correlation index (CCI) analysis was used to discriminate between mass spectra differences of the two cell types, classifying all 50 *C. parapsilosis* clinical isolates, included in the study, after only 3-h of testing, in high or low biofilm producers. All high (*n* = 25) or low (*n* = 25) biofilm producers had, according to CCI mass spectra comparison values, higher or lower than one CCI ratios, which were obtained by dividing the CCI_suspended cells_ by the CCI_attached cells_. In conclusion, the BIOF–HILO assay allows a rapid categorization of *C. parapsilosis* clinical isolates in high or low biofilm producers. This information, if timely provided to physicians, may improve treatment outcomes in patients with *C. parapsilosis* candidemia.

## Introduction

*Candida* species are able to adhere and form a biofilm on abiotic surfaces, such as on indwelling medical devices (e.g., vascular or urinary catheters), prosthetic heart valves, and joint replacements. This ability may be responsible for the high mortality attributed to *Candida* infections in healthcare settings (Tumbarello et al., 2007; Rajendran et al., 2016). Key factors favoring colonization and/or infection by biofilm-forming *Candida* species include high biofilm cell concentration, altered expression of cell ergosterol biosynthesis genes, and extracellular polymeric substances in the biofilm matrix (Finkel and Mitchell, 2011; Taff et al., 2013; Zarnowski et al., 2016). Notably, sessile and planktonic cells within *Candida* biofilms are different in their antifungal drug susceptibility profiles (Ramage et al., 2012), because biofilm growth would prompt the sessile cells to acquire a reduced susceptibility against antifungal agents (Silva et al., 2017).

During the last decade, *Candida parapsilosis* or *Candida glabrata* are on first and second position of non-*Candida albicans Candida* (NCAC) species isolated from patients with symptomatic bloodstream infection causing increased candidemia incidence (Guinea, 2014). *C. parapsilosis* exhibits the highest capability of adhering to abiotic surfaces among NCAC species (including *Candida tropicalis*) (Cavalheiro and Teixeira, 2018) and, consequently, this species is considered the most causative agent for catheter-related infections (Bouza et al., 2014). High (and/or moderate) biofilm formation levels were a significant risk factor for in-hospital mortality in *C. parapsilosis* candidemia (Soldini et al., 2018). Therefore, rapid information on high or low biofilm-forming *C. parapsilosis* isolates would aid to improve treatment outcomes in infected patients (Sanguinetti and Posteraro, 2016).

High or low biofilm-producing *C. parapsilosis* isolates differ regarding attached or suspended cells in the early *in vitro* adhesion phase of biofilm formation (Pannanusorn et al., 2014). This difference is beneficial to discriminate between the two isolate types after their 3-h adhesion. With this in mind, we developed the BIOF–HILO assay, which uses MALDI–TOF mass spectrometry (MS) technology coupled with the composite correlation index (CCI) analysis of protein profiles, allowing a rapid (i.e., 3-h) identification of high- or low-biofilm-forming *C. parapsilosis* isolates. Specifically, we performed a quantitative comparison of the mass spectra obtained from attached and suspended *C. parapsilosis* isolates’ cells during the initial *in vitro* biofilm formation. The BIOF–HILO assay and MALDI–TOF MS-based identification assay are substantially similar when comparing isolates’ spectra. However, we did not employ any reference database of *C. parapsilosis* mass spectra for comparison purposes in this assay.

## Materials and Methods

### Study Organisms

Fifty *C. parapsilosis sensu stricto* (hereafter referred to as *C. parapsilosis*) isolates collected from single patient candidemia episodes at the Fondazione Policlinico Universitario Agostino Gemelli IRCCS in Rome, Italy, were included in the study. The institutional Ethics Committee approved the study (no. 1401/16). No informed consent from patients was requested because testing was performed only on anonymized frozen isolates. These isolates had been characterized as high (*n* = 25) or low (*n* = 25) biofilm producers, i.e., as HP or LP, respectively, using the crystal-violet binding assay (Soldini et al., 2018). Isolates grew as biofilms in 96-well microtiter plates at 37°C for 24-h, starting from a standardized suspension (1 × 10^6^ cells/ml) in RPMI 1640 broth medium (Sigma-Aldrich, St. Louis, MO, United States). As measured by the crystal violet retention, the optical density at 540 nm (OD_540 nm_) values were >1.17 for HP isolates and <0.44 for LP isolates, according to the OD_540 nm_ cutoff values (<0.44, 0.44–1.17, and >1.17) used to classify *Candida* species isolates as low, moderate, and high biofilm level producers, respectively (Soldini et al., 2018).

### MALDI–TOF MS Assay

Preliminarily, one HP and one LP *C. parapsilosis* isolate at different time points (1.5, 3, 4.5, 6, 8, and 24 h) were studied using biological (i.e., three from repeated cultivations on different days) and technical (i.e., two from each same cultivation) isolate replicates, obtained as described below. These experiments allowed us to find the optimal time at which the differences between the HP and LP isolates’ mass spectra could be correctly detected (data not shown).

Upon optimization of the experimental parameters, all the 50 *C. parapsilosis* isolates were blindly tested with the BIOF–HILO assay. First, frozen isolate stocks were sub-cultured on Sabouraud dextrose agar (SDA) plates. Then, the isolates were grown in yeast extract–peptone–dextrose broth, under agitation at 150 rpm, overnight at 37°C. For each isolate, cells were harvested, washed twice with 0.15 M phosphate-buffered saline (PBS; pH 7.2, Ca^2+^- and Mg^2+^-free), and were used to obtain a 2-McFarland suspension (equivalent to ∼0.5 × 10^7^ cells/ml) in RPMI 1640 broth. After 3-h incubation in a 24-well cell culture plate, both the suspended (i.e., harvested by taking all the supernatant) and attached (i.e., harvested by scraping the well after addition of 200 μl of water) cells from each well were collected in two separate Eppendorf tubes, centrifuged, and twice washed in water. Randomly, a 0.1-ml aliquot of *C. parapsilosis* cells was plated in triplicate onto SDA and, then, the plates were read after 24-h incubation at 37°C for the CFU/ml determination. Expectedly, the number of cells attached to the microplate wells after 3-h was substantially higher than the number of cells suspended (i.e., non-attached) in HP isolates rather than in LP isolates (data not shown).

For MALDI–TOF MS analysis, the protein cell content was extracted from the 50 *C. parapsilosis* isolates, according to the fast formic-acid protocol previously developed (De Carolis et al., 2014b). The RNase B solution (1 mg/ml; Sigma-Aldrich, Milan, Italy) was used as an internal standard, which was added to each sample before MALDI–TOF mass spectra acquisition. Samples were spotted in duplicate on a MALDI polished target plate and measurements were performed with an MBT Smart mass spectrometer (Bruker Daltonics, Bremen, Germany) after calibration with a Bacterial Test Standard (Bruker Daltonics). The α-cyano-4-hydroxycinnamic acid (HCCA) matrix was used for sample crystallization. Spectra for each sample were generated from 500 laser shots acquired in automatic mode (100 laser shots at five different spot positions), and analyzed in a 3000–7000 Da range by dividing each spectrum into four intervals of the same size through the CCI tool of the MALDI Biotyper^TM^ system (Bruker Daltonics). The mass spectra obtained from each isolate’s suspended or attached cells and from the RNase B alone were compared with each other using the MALDI Biotyper 3.1 software by the CCI analysis (De Carolis et al., 2012), and were automatically visualized in a CCI matrix view.

### Organism Categorization

For comparison analysis, the MALDI–TOF mass spectrum profiles of the suspended or attached cells from each isolate’s biological replicates were matched against the internal standard’s mass spectrum (unpaired *t* test; *p* < 0.05). Then, each isolate was categorized as HP or LP if the CCI obtained by matching the internal standard’s profile vs. the suspended cells’ profile (CCI_susp_) was higher (i.e., in the case of HP) or lower (i.e., in the case of LP) than the CCI obtained by matching the internal standard’s profile vs. the attached cells’ profile (CCI_attach_), respectively. To take variation in the spectral profiles between HP and LP isolates into account CCI ratios were used, which were obtained by dividing the CCI_susp_ by the CCI_attach_ (see Supplementary Table S1). Accordingly, an isolate was defined as HP if the CCI_susp_/CCI_attach_ ratio was higher than one or as LP if the CCI_susp_/CCI_attach_ ratio was lower than one. Results were visualized as scatter plots using GraphPad Prism version 8.0.0 (GraphPad Software, Inc., La Jolla, CA, United States).

## Results and Discussion

**FIGURE 1:**
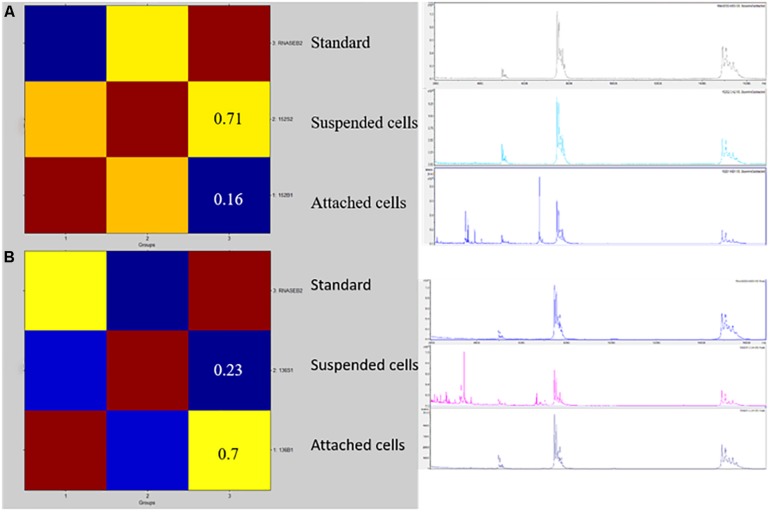
A CCI matrix based visualization for two representative isolates of HP **(A)** and LP **(B)** *C. parapsilosis*. Reddish colors indicate a close relationship between the isolates’ mass spectrum profiles. The CCI values refer to the suspended or attached cell profiles matched against the RNase-B internal standard’s profile, respectively. The right picture shows the row spectra profiles of the internal standard and the suspended or attached isolates’ cells used to create the CCI matrix.

**FIGURE 2:**
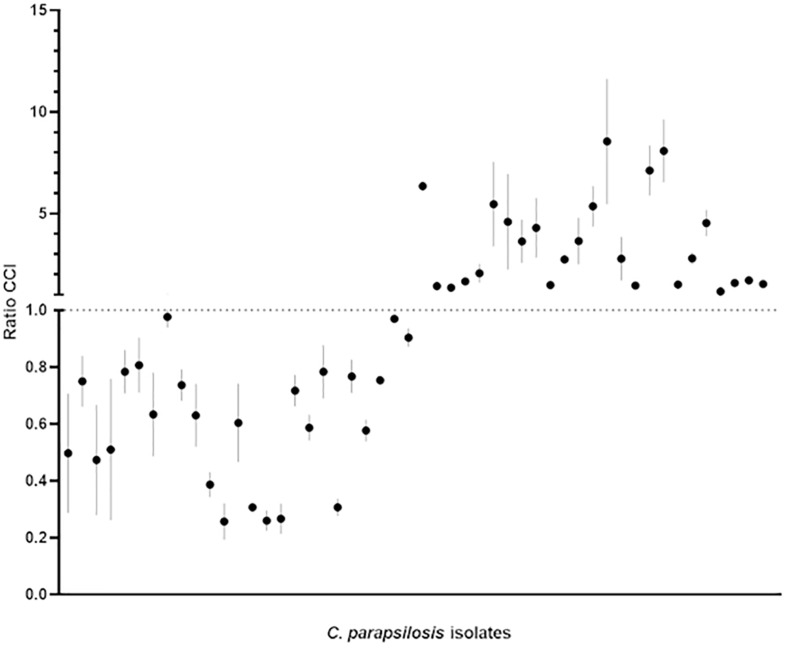
Categorization of the 50 *C. parapsilosis* isolates (25 HP and 25 LP) included in the study based on the CCI_susp_/CCI_attach_ ratio (for details, see Supplementary Table S1). Bars indicate the minimum and maximum CCI ratio obtained for each of the three biological replicates of each isolate, with the mean value marked as a dark dot. According to the discriminatory power of the BIOF–HILO assay, the HP and LP isolates settled in the upper or the lower part of the plot, respectively.

**FIGURE 3:**
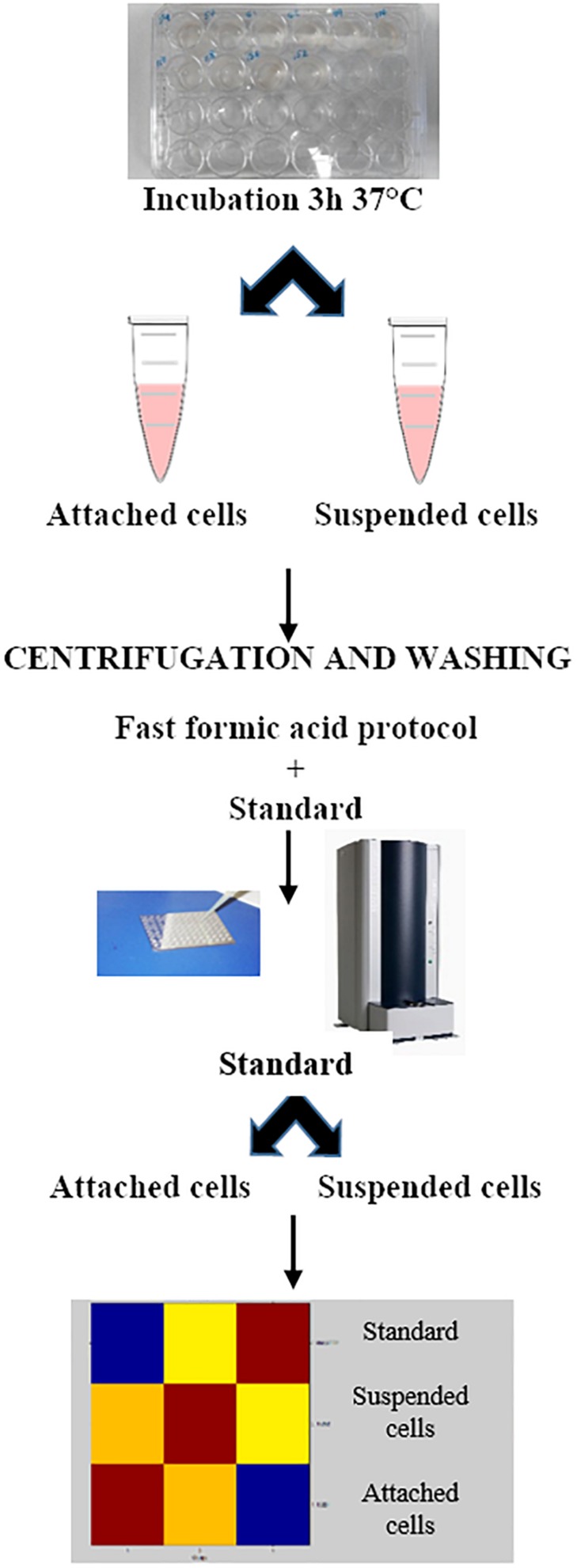
The BIOF–HILO assay workflow.

Figures 1, 2 show the BIOF–HILO assay to discriminate between HP and LP *C. parapsilosis* isolates. Specifically, Figure 2 illustrates that the BIOF–HILO assay discriminated all clinical isolates (*n* = 50) within 3-h in line with the biofilm levels (high or low) as previously established by Soldini et al. (2018) (see also Supplementary Table S1). Figure 3 shows a comprehensive representation (from the sample to the result) of the BIOF–HILO assay. Based on our findings, BIOF–HILO assay, testing adhesions after 3-h, provides a substitute for the test after 24-h to measure the biofilm formed by clinical *C. parapsilosis* isolates. Consistent with our data, Pannanusorn et al. (2014) showed in an *in vitro* study that the initial adhesion to plastic surfaces (e.g., plate wells) after 3-h correlates with the different biofilm phenotypes displayed by clinical *C. parapsilosis* isolates, which in turn reflect their capacity to form high or low biofilm.

Our findings provide further insights into the clinical diagnostic possibilities of MALDI–TOF MS shown for *Candida* species (including *C. parapsilosis*). These possibilities encompass the *in vitro* assays for fungal identification, susceptibility/resistance detection, and typing, particularly for emerging *Candida* species (De Carolis et al., 2014a; Dhieb et al., 2015; Vella et al., 2017; Bao et al., 2018; Paul et al., 2018; Vatanshenassan et al., 2018, 2019). Very few studies, to date, tempted to apply the MALDI–TOF MS technology to the direct detection of biofilm-forming isolates of *Candida* species. Kubesová et al. (2012) used MALDI–TOF MS with intact cells and with sinapinic acid as a MALDI matrix to distinguish rapidly and reliably between two biofilm-negative and two biofilm-positive strains of *C. parapsilosis* (and *Candida metapsilosis*). Mlynáriková et al. (2016) used MALDI–TOF MS with ethanol/formic-acid extracted cells to differentiate between 12 biofilm-positive and 9 biofilm-negative *C. parapsilosis* strains using different MALDI matrices (HCCA, ferulic acid, etc.) and experimental conditions (cultivation times or media). However, the authors failed to group reliably the strains in two clusters within a MALDI–TOF mass spectra-based dendrogram, because mass spectra variations seemed to limit the reproducibility of the method.

We focused on the early development phase of the *C. parapsilosis* biofilm with MALDI–TOF MS analysis. Furthermore, we employed an automated CCI matrix-based algorithm facilitating the relatedness analysis between the mass spectra from HP or LP *C. parapsilosis* isolates. This algorithm allowed us to interpret objectively the MALDI–TOF MS analysis results and to achieve a reliable separation between the mass spectra profiles derived from experimental samples such as the attached or suspended cells of *C. parapsilosis* isolates, as a fingerprint matching technique previously described (De Carolis et al., 2012). We assessed the extent of this separation between the mass spectra of HP or LP *C. parapsilosis* isolates by quantifying their differences in mass peaks with respect to the RNase-B internal standard. In this context, we chose a molecular mass range analysis of 3000–7000 Da, which excluded the portion of the mass spectrum profile where only RNase-B mass peaks are detectable but allowed to assess the isolates’ mass peaks within the lower range.

MALDI–TOF MS assays are helpful to identify directly microbial organisms from clinical samples (Rodríguez-Sánchez et al., 2019). However, at least for *Candida* organisms, growing the isolates before the spectral profiles MALDI–TOF MS analysis is still a fundamental step when testing isolates for their capability of biofilm formation. Our assay seems to be particularly promising as it offers the possibility to combine MALDI–TOF MS-based identification with biofilm formation testing. In the latter case, only a short-incubation is required prior to testing; this, coupled with an unbiased capability of classifying the isolates, implies the assay to be advantageous compared with the conventional (Sanguinetti and Posteraro, 2016) or previously developed MALDI–TOF MS-based (Mlynáriková et al., 2016) biofilm detection assays. The rapid and inexpensive protein spectra acquisition using MALDI–TOF MS has led the technology to a standard tool in clinical settings. Currently, MALDI–TOF MS allows obtaining reliable identification and antimicrobial susceptibility testing results for the microbial pathogens within one working shift (Rodríguez-Sánchez et al., 2019). As these results are crucial for timely administration of effective antimicrobial treatment, the simultaneous additional information on the status of biofilm formation by pathogens in a quick manner may therefore enhance the likelihood that the administered antimicrobial treatment will be efficacious.

In conclusion, we ideated the BIOF–HILO assay with the purpose to provide a more rapid identification of *C. parapsilosis* isolates with high or low capacity of biofilm formation compared to the methods currently used in the clinical mycology laboratory (Sanguinetti and Posteraro, 2016). However, further studies using a larger number of *C. parapsilosis* isolates are needed, which can validate the present findings and additionally establish whether the BIOF–HILO assay may have in the near future a place in laboratory diagnostics.

## Data Availability

All datasets generated for this study are included in the manuscript and/or the Supplementary Files.

## Author Contributions

All authors listed have made a substantial, direct and intellectual contribution to the work, and approved it for publication. In particular, EDC conceived the study, participated in the experimental conduction of the study, and drafted the manuscript. SS, MLR, and FN performed the experiments and interpreted the data. BP and MS participated in the design and coordination of the study, and critically revised the manuscript.

## Conflict of Interest Statement

The authors declare that the research was conducted in the absence of any commercial or financial relationships that could be construed as a potential conflict of interest.

## Supplementary Material

TABLE S1 Experimental data on MALDI–TOF MS analysis and biofilm formation for the HP or LP *C. parapsilosis* isolates included in the study.
