# Supplementary material for: BIOF–HILO Assay: A New MALDI–TOF Mass Spectrometry Based Method for Discriminating Between High- and Low-Biofilm-Producing Candida parapsilosis Isolates
Source: Front Microbiol. 2019 Aug 30;10:2046. doi: 10.3389/fmicb.2019.02046 (PMC6728890; doi:10.3389/fmicb.2019.02046)
Supplement: TABLE S1 — Experimental data on MALDI–TOF MS analysis and biofilm formation for the HP or LP C. parapsilosis isolates included in the study. [file Table_1.DOCX]

| **LP Isolate^a^** | **C.V (OD)_540 nm_^b^** | **St. vs Attach.^c^** | **St. vs Susp.^d^** | **Ratio^e^** |
| --- | --- | --- | --- | --- |
| 147 | 0.07 | 0.83 | 0.29 | 0.35 |
|  |  | 0.84 | 0.20 | 0.23 |
|  |  | 0.42 | 0.38 | 0.91 |
| 1 | 0.36 | 0.37 | 0.21 | 0.58 |
|  |  | 0.80 | 0.70 | 0.88 |
|  |  | 0.76 | 0.60 | 0.79 |
| 2 | 0.19 | 0.47 | 0.13 | 0.28 |
|  |  | 0.64 | 0.18 | 0.28 |
|  |  | 0.66 | 0.57 | 0.86 |
| 5 | 0.12 | 0.77 | 0.77 | 1.00 |
|  |  | 0.47 | 0.15 | 0.33 |
|  |  | 0.42 | 0.08 | 0.20 |
| 10 | 0.27 | 0.83 | 0.77 | 0.93 |
|  |  | 0.45 | 0.31 | 0.68 |
|  |  | 0.45 | 0.33 | 0.74 |
| 11 | 0.25 | 0.58 | 0.37 | 0.63 |
|  |  | 0.76 | 0.73 | 0.96 |
|  |  | 0.32 | 0.27 | 0.83 |
| 14 | 0.18 | 0.67 | 0.56 | 0.84 |
|  |  | 0.83 | 0.29 | 0.35 |
|  |  | 0.28 | 0.19 | 0.71 |
| 19 | 0.26 | 0.73 | 0.76 | 1.04 |
|  |  | 0.36 | 0.36 | 0.98 |
|  |  | 0.38 | 0.35 | 0.91 |
| 29 | 0.16 | 0.77 | 0.64 | 0.83 |
|  |  | 0.77 | 0.57 | 0.74 |
|  |  | 0.32 | 0.21 | 0.64 |
| 30 | 0.06 | 0.77 | 0.58 | 0.75 |
|  |  | 0.81 | 0.59 | 0.73 |
|  |  | 0.56 | 0.23 | 0.41 |
| 136 | 0.08 | 0.94 | 0.40 | 0.43 |
|  |  | 0.95 | 0.41 | 0.43 |
|  |  | 0.97 | 0.29 | 0.30 |
| 39 | 0.12 | 0.58 | 0.22 | 0.38 |
|  |  | 0.66 | 0.11 | 0.17 |
|  |  | 0.68 | 0.15 | 0.22 |
| 40 | 0.006 | 0.83 | 0.59 | 0.72 |
|  |  | 0.84 | 0.64 | 0.76 |
|  |  | 0.85 | 0.28 | 0.33 |
| 45 | 0.11 | 0.95 | 0.32 | 0.33 |
|  |  | 0.95 | 0.29 | 0.31 |
|  |  | 0.95 | 0.27 | 0.28 |
| 47 | 0.16 | 0.63 | 0.18 | 0.29 |
|  |  | 0.65 | 0.20 | 0.30 |
|  |  | 0.70 | 0.14 | 0.19 |
| 34 | 0.2 | 0.90 | 0.21 | 0.23 |
|  |  | 0.64 | 0.24 | 0.37 |
|  |  | 0.97 | 0.19 | 0.20 |
| 151 | 0.1 | 0.71 | 0.44 | 0.62 |
|  |  | 0.68 | 0.49 | 0.72 |
|  |  | 0.59 | 0.48 | 0.81 |
| 50 | 0.08 | 0.25 | 0.17 | 0.65 |
|  |  | 0.28 | 0.17 | 0.61 |
|  |  | 0.28 | 0.14 | 0.50 |
| 51 | 0.08 | 0.84 | 0.78 | 0.93 |
|  |  | 0.22 | 0.18 | 0.81 |
|  |  | 0.33 | 0.20 | 0.61 |
| 52 | 0.15 | 0.52 | 0.18 | 0.35 |
|  |  | 0.66 | 0.17 | 0.25 |
|  |  | 0.51 | 0.16 | 0.32 |
| 53 | 0.07 | 0.54 | 0.47 | 0.87 |
|  |  | 0.61 | 0.41 | 0.67 |
|  |  | 0.54 | 0.41 | 0.76 |
| 54 | 0.08 | 0.66 | 0.39 | 0.58 |
|  |  | 0.65 | 0.42 | 0.64 |
|  |  | 0.68 | 0.34 | 0.51 |
| 57 | 0.29 | 0.64 | 0.48 | 0.75 |
|  |  | 0.64 | 0.47 | 0.73 |
|  |  | 0.63 | 0.49 | 0.78 |
| 61 | 0.18 | 0.49 | 0.47 | 0.97 |
|  |  | 0.48 | 0.46 | 0.96 |
|  |  | 0.49 | 0.48 | 0.98 |
| 62 | 0.3 | 0.51 | 0.47 | 0.93 |
|  |  | 0.52 | 0.43 | 0.84 |
|  |  | 0.52 | 0.49 | 0.94 |
| **HP Isolate^a^** | **C.V (OD)_540 nm_^b^** | **St. vs Attach.^c^** | **St. vs Susp.^d^** | **Ratio^e^** |
| 152 | 2.3 | 0.14 | 0.91 | 6.37 |
|  |  | 0.13 | 0.86 | 6.65 |
|  |  | 0.15 | 0.9 | 6.01 |
| 4 | 2.6 | 0.34 | 0.50 | 1.49 |
|  |  | 0.54 | 0.76 | 1.41 |
|  |  | 0.28 | 0.38 | 1.36 |
| 8 | 2.45 | 0.63 | 0.91 | 1.44 |
|  |  | 0.71 | 0.92 | 1.30 |
|  |  | 0.33 | 0.43 | 1.30 |
| 15 | 2.8 | 0.22 | 0.38 | 1.72 |
|  |  | 0.50 | 0.83 | 1.65 |
|  |  | 0.25 | 0.4 | 1.60 |
| 16 | 2 | 0.1 | 0.29 | 2.95 |
|  |  | 0.24 | 0.22 | 1.58 |
|  |  | 0.52 | 0.85 | 1.63 |
| 17 | 1.3 | 0.14 | 0.93 | 6.49 |
|  |  | 0.57 | 0.83 | 1.46 |
|  |  | 0.1 | 0.84 | 8.42 |
| 20 | 1.2 | 0.71 | 0.89 | 1.25 |
|  |  | 0.27 | 0.92 | 3.42 |
|  |  | 0.1 | 0.91 | 9.10 |
| 28 | 1.2 | 0.53 | 0.9 | 1.70 |
|  |  | 0.24 | 0.92 | 3.86 |
|  |  | 0.18 | 0.97 | 5.32 |
| 32 | 1.16 | 0.21 | 0.82 | 3.89 |
|  |  | 0.45 | 0.89 | 1.98 |
|  |  | 0.1 | 0.7 | 7.00 |
| 37 | 1.19 | 0.47 | 0.78 | 1.66 |
|  |  | 0.19 | 0.27 | 1.42 |
|  |  | 0.24 | 0.32 | 1.33 |
| 43 | 1.19 | 0.1 | 0.28 | 2.84 |
|  |  | 0.1 | 0.26 | 2.60 |
|  |  | 0.09 | 0.26 | 2.78 |
| 48 | 1.78 | 0.09 | 0.38 | 4.10 |
|  |  | 0.53 | 0.79 | 1.49 |
|  |  | 0.07 | 0.37 | 5.34 |
| 55 | 1.83 | 0.07 | 0.49 | 7.07 |
|  |  | 0.08 | 0.43 | 5.36 |
|  |  | 0.08 | 0.29 | 3.63 |
| 60 | 2.17 | 0.08 | 0.40 | 5.06 |
|  |  | 0.07 | 0.41 | 5.90 |
|  |  | 0.03 | 0.44 | 14.67 |
| 126 | 0.99 | 0.46 | 0.86 | 1.87 |
|  |  | 0.5 | 0.78 | 1.56 |
|  |  | 0.09 | 0.44 | 4.89 |
| 146 | 0.66 | 0.38 | 0.65 | 1.71 |
|  |  | 0.5 | 0.62 | 1.24 |
|  |  | 0.38 | 0.53 | 1.39 |
| 64 | 0.45 | 0.09 | 0.56 | 5.86 |
|  |  | 0.1 | 0.57 | 5.93 |
|  |  | 0.04 | 0.39 | 9.56 |
| 65 | 0.34 | 0.08 | 0.73 | 9.63 |
|  |  | 0.07 | 0.71 | 9.61 |
|  |  | 0.12 | 0.6 | 5.00 |
| 85 | 0.28 | 0.55 | 0.87 | 1.58 |
|  |  | 0.55 | 0.84 | 1.53 |
|  |  | 0.57 | 0.8 | 1.40 |
| 94 | 0.2 | 0.11 | 0.36 | 3.23 |
|  |  | 0.13 | 0.28 | 2.25 |
|  |  | 0.13 | 0.39 | 2.89 |
| 98 | 0.35 | 0.18 | 0.58 | 3.26 |
|  |  | 0.11 | 0.61 | 5.31 |
|  |  | 0.13 | 0.65 | 5.01 |
| 99 | 0.5 | 0.43 | 0.48 | 1.10 |
|  |  | 0.47 | 0.51 | 1.09 |
|  |  | 0.4 | 0.51 | 1.29 |
| 116 | 0.23 | 0.53 | 0.83 | 1.57 |
|  |  | 0.5 | 0.9 | 1.80 |
|  |  | 0.54 | 0.74 | 1.37 |
| 117 | 0.32 | 0.52 | 0.91 | 1.75 |
|  |  | 0.54 | 0.92 | 1.70 |
|  |  | 0.55 | 0.91 | 1.65 |
| 150 | 0.33 | 0.46 | 0.7 | 1.52 |
|  |  | 0.49 | 0.69 | 1.41 |
|  |  | 0.44 | 0.72 | 1.64 |

**Supplementary table 1.** ^a^ C. parapsilosis isolate number for LP or HP are indicated.

*^b^ C. parapsilosis* isolates were categorized using optical density (OD) _540 nm_ cut-off values <0.44 and >1.17 for biofilm LP or HP strains, respectively.

^c^ Composite correlation index (CCI) obtained by matching of standard molecule profile vs attached cells profile.

^d^ Composite correlation index (CCI) obtained by matching of standard molecule profile vs suspended cells profile.

^e^ Ratio between ^d^ and ^c^ CCI.
